# Effect of epidural labor analgesia on maternal and infant outcomes in parturients with gestational diabetes mellitus—A prospective cohort study

**DOI:** 10.3389/fped.2022.1022291

**Published:** 2022-12-12

**Authors:** Gehui Li, Xiaofei Qi, Xuhong Tan, Mingguang Wu, Hao Wang, Ping Wen, Xiaolei Huang, Yuantao Li

**Affiliations:** ^1^ Department of Anesthesiology, Shenzhen Maternity and Child Healthcare Hospital, Southern Medical University, Shenzhen, China; ^2^ Department of Food Safety, Market Supervision Administration of Shenzhen Municipality, Shenzhen, China; ^3^ Department of Science and Education, Shenzhen Maternity and Child Healthcare Hospital, Southern Medical University, Shenzhen, China

**Keywords:** epidural labor analgesia, gestational diabetes mellitus (GDM), neonatal hypoglycemia, infant outcomes, maternal outcomes

## Abstract

**Background:**

The occurrence of gestational diabetes mellitus (GDM) is caused by a variety of factors and associated with increased risks of several adverse outcomes for both mothers and infants. However, the effects of epidural labor analgesia in parturients with GDM on maternal and infant outcomes have not been characterized.

**Methods:**

According to parturients' choice, they were divided into the epidural group (*n* = 133) and no epidural (control) group (*n* = 135). Data for relative variables in the perinatal period were collected, and the potential associations of epidural labor analgesia with infant outcomes were analyzed by univariate analysis and multivariate logistic regression analyses.

**Results:**

The rate of neonatal admission to the neonatal intensive care unit (NICU) for hypoglycemia was higher in the epidural group (7.52%) than in the control group (1.48%; *P* < 0.05). Epidural labor analgesia and drug-based diabetes control were independent predictors of the rate of neonate transfer to the NICU for hypoglycemia.

**Conclusion:**

Epidural labor analgesia was associated with an increased risk of neonatal transfer to the NICU for hypoglycemia. Thus, monitoring of neonatal blood glucose levels after administration of epidural labor analgesia in parturients with GDM may be beneficial.

**Trial registration:** The study was registered in the China Clinical Registration Center (Registration No. ChiCTR-OOC-17013164, Registered on 30 October 2017).

## Background

Gestational diabetes mellitus (GDM) is the most common medical complication of pregnancy. It is associated with adverse maternal and infant outcomes (1). The total incidence of GDM in mainland China is 14.8% (95% confidence interval 12.8%–16.7%) (2). GDM is a pregnancy complication of abnormal glucose metabolism that can be caused by a variety of factors (3) and has many adverse effects on mothers and children in both the short and long terms, such as pregnant hypertension, prematurity, macrosomia, intrauterine death and perinatal asphyxia, and neonatal hypoglycemia (4).

Labor pain induces a neuroendocrine response to stress and usually induces a further increase in blood glucose levels (5, 6). In parturients with GDM, this can aggravate blood glucose fluctuation, causing further metabolic disorder that can have adverse effects on the fetus. Accordingly, researchers have investigated the benefits of labor analgesia in these patients and reported that it can relieve the stress response and cortisol levels in parturients (7, 8).

Although these different correlations have been reported, few clinical data confirming the effect of epidural labor analgesia on maternal and infant outcomes are available. Therefore, this prospective observational cohort study aimed to explore whether epidural labor analgesia is associated with maternal and infant outcomes, and the potential associations of epidural labor analgesia with infant outcomes were analyzed. Although a high-quality randomized controlled trial (RCT) is needed, randomizing healthy pregnant women is ethically difficult and may lead to over-exclusion of the normal population. Propensity score matching (PSM) is a statistical method to collect data and minimize selective bias generated by patients' backgrounds. Many studies have reported that PSM produces results similar to those of RCTs (9).

## Methods

### Study design and participants

Parturients were recruited in Shenzhen Maternity and Child Healthcare Hospital from January 2018 to November 2019. Inclusion criteria: GDM was diagnosed according to IADPSG 2010 diagnostic criteria (10); gestational age ≥35 weeks; successful entry into labor rather than failure in induction of labor (artificial methods were required to induction and induction of labor failed), and single fetal head position. The exclusion criteria included: declined consent, and contraindications to epidural anesthesia (such as central nervous system diseases, coagulopathy, shock, systemic or puncture site infection, non-cooperation, etc.). Parturients were sent to the delivery room when cervical dilation reached 3 cm (primipara) or 2 cm (multipara). All parturients provided written informed consent to participation in the study, and epidural labor analgesia was carried out entirely according to the parturients' wishes.

The institutional management of diabetes in labor protocols was based on the guidelines of American Diabetes Association (2018), and maternal blood glucose control in pregnancy was based on hbA1C measurement (normal <6%) (11).

The research proposal was approved by the Ethics Committee of Shenzhen Maternal and Child Healthcare Hospital (Approval No. SZFY2017102095).

### Data collection

The following data were obtained through medical records reviews, questionnaires, and oral interviews: basic demographic data (age, height, weight, BMI, gestational age, and parity), obstetric history (adverse pregnancy history and obstetric complications), pregnancy-related information (source of health information during pregnancy, planned or unplanned pregnancy), lifestyle habits (smoking, drinking and long-term medication), methods of blood glucose control (diet or medication) and blood glucose levels.

The analgesic effect in parturients was assessed using the Numerical Rating Scale (NRS), with patients rating their pain on a scale from 0 to 10 (12). The progress of labor, delivery outcomes, and adverse reactions (itching, dizziness, chills, nausea, vomiting, urinary retention) were recorded.

The following neonatal clinical data were recorded: sex, body weight, 1- and 5-min Apgar scores, and heel blood glucose levels at 1, 2 and 3 h after birth. A blood glucose level less than 2.6 mmol/L is considered the limit value for clinical treatment of hypoglycemia (13). Therefore, the blood glucose level was rechecked if <2.6 mmol/L, and a repeated abnormal level prompted transfer to the neonatal intensive care unit (NICU) for treatment of hypoglycemia.

### Epidural labor analgesia

The L2–3 epidural space was selected for epidural analgesia. Experimental doses of lidocaine were injected to rule out intravascular and subarachnoid catheterization, followed by 10 ml loading dose (0.125% ropivacaine and 0.4 μg/ml sufentanil). Then the patient-controlled epidural analgesia (PCEA) pump was connected (0.1% ropivacaine + sufentanil 0.4 μg/ml, background dose 5 ml/h, bulos dose 5 ml, locking time 15 min). If the parturients' NRS score >4, the anesthetic nurse gave an additional 5 ml of remedial medication. NRS scores at baseline, 5 cm and 10 cm of cervical dilation were recorded. Parturients' blood pressure was monitored, and patients received norepinephrine if hypotension (>30% lower than baseline) occurred and atropine if bradycardia (heart rate <60 bpm) occurred.

### Statistical analysis

The primary indicator in this study was maternal and infant outcomes, especially the rate of admission to the NICU for hypoglycemia. For the calculation of the independent sample size of the two groups, because the rate of labor analgesia in our hospital in 2017 was 48.5%, we assumed the same numbers of patients would be included in the two groups. According to the preliminary experimental results, we assumed that the rate of admission to the NICU for hypoglycemia in the epidural labor analgesia group would be 51%. In the non-epidural labor analgesia group, the incidence was 29%. For 90% power and 0.05 two-tailed significance, Power Analysis and Sample Size (PASS) 2011 software (NCSS, LLC) was used to calculate the sample size required for each group to be 103. To make up for a 20% loss to follow-up, 124 patients were needed in each group.

SPSS 25.0 software was used for all statistical analyses. For quantitative data, if the data followed a normal distribution, mean ± standard deviation values were used, and the significance of differences between groups was tested by *t* test. The median (upper quartile, lower quartile) values were used to describe data with a non-normal distribution, and the significance of the differences between groups was tested by rank sum test. The use rate or composition ratio (%) of count data was described, and *χ*^2^ test or Fisher's exact probability method was used for comparison between groups. The variables with statistical significance on univariate analysis were introduced into logistic regression analysis to analyze the relationships of maternal outcomes as well as NICU transfer for hypoglycemia with epidural labor analgesia, age, and BMI. Epidural analgesia was taken as the dependent variable, and a statistically significant difference between the two groups was taken as the independent variable. Using 1 : 1 nearest neighbor matching, the caliper value is set to 0.02. PSM was performed to analyze the significant relationship between maternal outcome and NICU transfer due to hypoglycemia and other variables. A two-tailed *P* value <0.05 indicated a significant difference.

## Results

### Demographic and clinical characteristics of enrolled parturients

**Figure 1:**
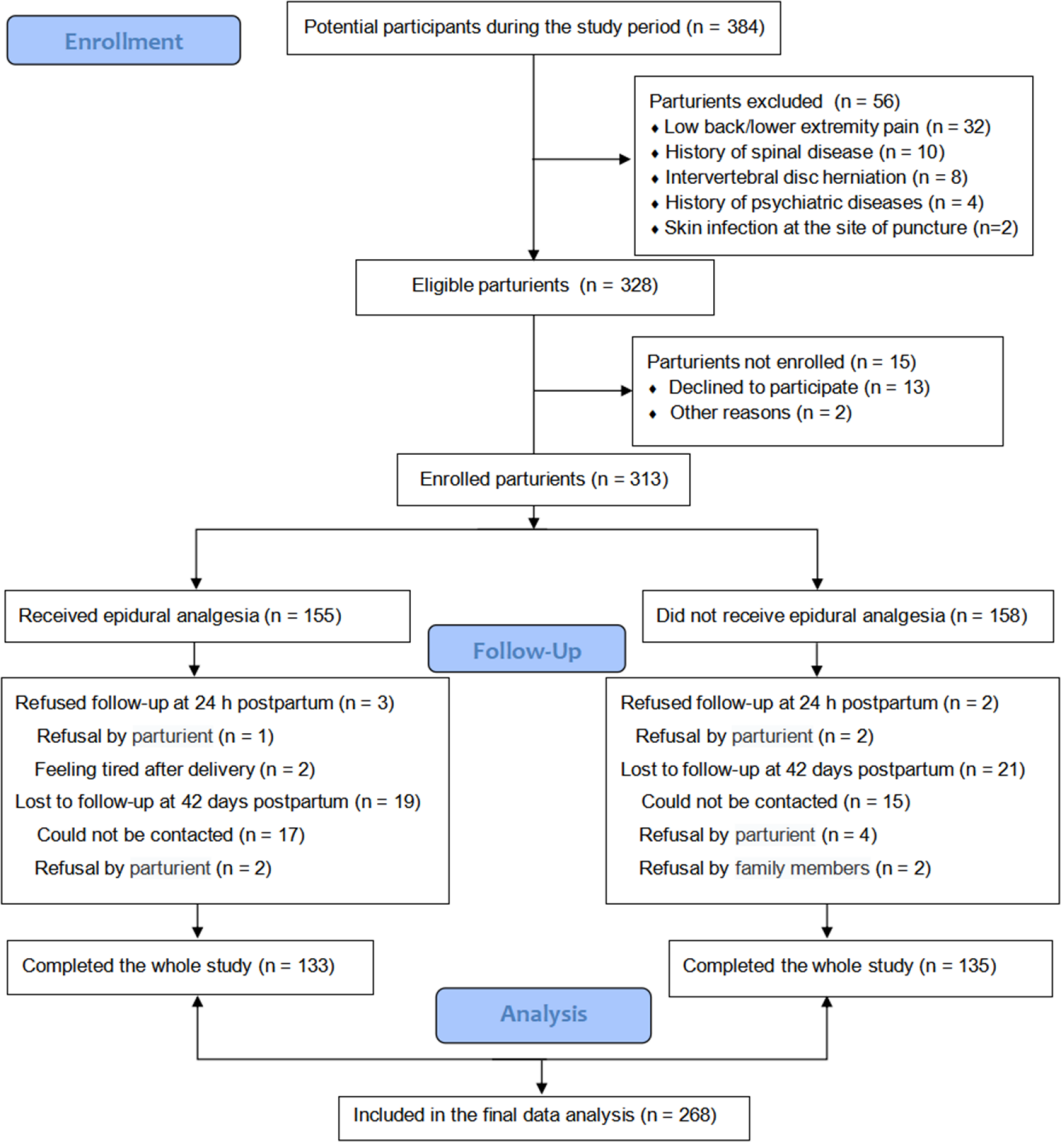
Flow chart of patient enrollment.

Figure 1 shows a flow chart that outlines the study enrollment process. According to the inclusion and exclusion criteria, 328 eligible patients were selected and 56 patients were excluded. Of the 313 patients enrolled in the study, 45 were lost to follow-up at 24 h. Finally, 268 parturients completed all follow-up and were included in the analysis, of which 133 (49.63%) received epidural labor analgesia. Additionally, the baseline epidural rate was very even.

Table 1 shows the demographic data of the enrolled patients. The age and rate of abnormal pregnancy history in the control group were higher than those in the epidural group (*P* < 0.05). There were more multiparas in the control group and more primiparas in the epidural group (*P* = 0.000).

**Table 1:** Baseline demographic and obstetric characteristics of parturients who completed the study.

Characteristic	Epidural group (*n* = 133)	No epidural (control) group (*n* = 135)	*P*
Ages (years)	30.36 ± 3.79	32.75 ± 4.18	0.000
Height (cm)	159.14 ± 5.03	159.36 ± 5.05	0.712
Weight (kg)	62.10 ± 8.08	61.74 ± 10.79	0.761
Body mass index (kg/m^2^)	24.51 ± 2.87	24.29 ± 3.96	0.618
Gestational age at delivery (weeks)	38.98 ± 1.07	38.72 ± 1.49	0.106
**Gravidity**			
Primipara	89 (66.92%)	32 (23.70%)	0.000
Multipara	44 (33.08%)	103 (76.30%)	0.000
**Source of health knowledge during pregnancy**			
Routine obstetric examination	128 (96.24%)	133 (98.52%)	0.432
Maternity classes	100 (75.19%)	101 (74.81%)	0.944
Internet resources or books	126 (94.74%)	130 (96.30%)	0.537
**Maternal situational factors**			
Unplanned pregnancy	27 (20.30%)	32 (23.70%)	0.501
History of abnormal pregnancy^a^	29 (21.80%)	51 (37.78%)	0.004
Pregnancy with obstetric disease^b^	12 (9.02%)	14 (10.37%)	0.709
Cigarette, alcohol, long-term medication use during pregnancy	0 (0%)	1 (0.74%)	1.000
**Method for diabetes control**			
Diet	123 (92.48%)	121 (89.63%)	0.414
Medication	10 (7.52%)	14 (10.37%)	0.414
**Maternal blood glucose control during pregnancy (normal)**	111 (83.46%)	114 (84.44%)	0.826

Data are presented as mean ± SD, number of patients (percentage), or median (range).

BMI, body mass index; SD, standard deviation.

Comparisons were made using Student's *t*-test or Wilcoxon rank sum test for non-normally distributed variables or using Pearson's *χ*^2^ test and Fisher's exact test for proportions.

^a^ Abnormal pregnancy included embryo termination, fetal malformation, stillbirth, stillbirth history, postpartum hemorrhage, ectopic pregnancy, etc.

^b^ Obstetric diseases included pregnancy-induced hypertension syndrome, low free triiodothyronine and free thyroxine during pregnancy.

### Effect of epidural labor analgesia on pain relief, adverse events during delivery, and the incidence of cesarean delivery

The NRS pain score in the epidural analgesia group was significantly lower than that in the control group when cervical dilation was 5 cm and 10 cm (*P* = 0.000). The duration of the first and second stages of labor was longer in the epidural group than in the control group (*P* = 0.000). The incidence rates of pruritus, dizziness and urinary retention were higher in the epidural group than in the control group (Table 2).

**Table 2:** Perinatal variables of parturients who completed the study.

Variable	Epidural group (*n* = 133)	No epidural (control) group (*n* = 135)	*P*
**NRS score**			
Baseline	8.87 ± 1.58	8.85 ± 1.70	0.856
At cervical dilation 5 cm	3.34 ± 1.79	9.03 ± 1.83	0.000
At cervical dilation 10 cm	3.67 ± 1.79	9.19 ± 1.58	0.000
**Duration of labor (min)**			
Stage 1	596.38 ± 31.35	202.53 ± 13.79	0.000
Stage 2	52.05 ± 45.96	18.83 ± 20.02	0.000
Stage 3	9.66 ± 6.39	9.44 ± 5.77	0.859
**Adverse events during delivery**			
Itch	9 (6.77%)	2 (1.48%)	0.029
Dizzy	25 (18.80%)	13 (9.63%)	0.031
Nausea	7 (5.26%)	7 (5.19%)	0.977
Vomiting	9 (6.77%)	3 (2.22%)	0.072
Chills	27 (20.30%)	18 (13.33%)	0.127
Urinary retention	12 (9.02%)	2 (1.48%)	0.006
**Mode of delivery**			
Vaginal birth	132 (99.25%)	133 (98.52%)	1.000
Cesarean	1 (0.75%)	2 (1.5%)	1.000

Data are presented as mean ± SD, number of patients (percentage), or median (range).

NRS, numeric rating scale; Comparisons were made using Student's *t*-test or Wilcoxon rank sum test for non-normally distributed variables and using Pearson's *χ*^2^ test and Fisher's exact test for proportions.

Vaginal delivery was 99.25% in the epidural group (132/133) and 98.52% of parturients in the control group (133/135). No significant difference in the rate of vaginal delivery was observed between the two groups (Table 2).

### Effect of epidural labor analgesia on neonatal variables

When neonatal health outcomes were compared between the epidural group and control group, no significant differences in the infants' weight, sex, and 1-min and 5-min Apgar scores were observed between the groups (Table 3). However, the incidence of neonatal hypoglycemia (<2.6 mmol/L) was higher in the epidural group than in the control group at 2 h (6.77% [9/133] vs. 0.74% [1/135], *P* = 0.019) after delivery. Twenty-seven newborns were transferred to the NICU (1 for congenital malformation, 1 for congenital pericardial effusion, 9 for mild asphyxia, 1 for jaundice, 2 for low birth weight, 2 for maternal fever, 1 for macrosomia, and 10 for hypoglycemia) in the epidural group, and 14 newborns were transferred to the NICU (7 for mild asphyxia, 4 for low birth weight, 1 for anemia, and 2 for hypoglycemia) in the control group. The percentage of newborns who required NICU care for hypoglycemia was 7.52% (10/133) in the epidural group compared with only 1.48% (2/135) in the control group (*P* = 0.017; Table 3).

**Table 3:** Health outcomes for newborns of parturients enrolled in this study.

Neonatal outcomes	Epidural group (*n* = 133)	No epidural (control) group, (*n* = 135)	*P*
Neonatal weight (g)	3281 ± 401	3192 ± 466	0.095
Neonatal weight ≥3,500 g	35 (26.32%)	31 (22.96%)	0.524
**Neonatal gender**			
Male	79 (59.40%)	74 (54.81%)	0.448
Female	55 (41.35%)	61 (45.19%)	0.527
**Apgar score**			
1 min	10 (7–10)	10 (9–10)	0.921
5 min	10 (10–10)	10 (10–10)	0.345
**Heel blood glucose after birth**			
<2.6 mmol/L at 1 h	16 (11.54%)	8 (5.93%)	0.080
<2.6 mmol/L at 2 h	9 (6.77%)	1 (0.74%)	0.019
<2.6 mmol/L at 3 h	7 (5.26%)	2 (1.48%)	0.102
NICU admission after birth	27 (20.30%)	14 (10.37%)	0.024
NICU admission after birth for hypoglycemia treatment	10 (7.52%)	2 (1.48%)	0.017

Data are presented as number of patients (percentage), mean ± SD, or median (range). Comparisons were made using Student's *t*-test or Wilcoxon rank sum test for non-normally distributed variables or using Pearson's *χ*^2^ test and Fisher's exact test for proportions.

### Factors associated with neonate transfer to the NICU for hypoglycemia treatment

To further analyze the influence of epidural labor analgesia on the health of the newborn, we considered the rate of neonatal hypoglycemia requiring further treatment in the NICU as the risk index of neonatal hypoglycemia and regarded it as the dependent variable in univariate and multivariate analyses. Univariate logistic regression analysis identified two maternal and infant variables as significantly associated with the rate of neonatal hypoglycemia (*P* < 0.05): epidural analgesia and method of diabetes control (diet vs. medication). Multivariate logistic regression analysis further identified only one independent predictor, method of diabetes control (diet vs. medication) as a risk factor (odds ratio [OR], 5.277; 95% confidence interval [CI], 1.181–23.580; *P* < 0.05) that increased the risk of neonate transfer to the NICU for hypoglycemia treatment (Table 4).

**Table 4:** Results of univariate and multivariate analyses of factors associated with the rate of neonate transfer to the NICU for hypoglycemia treatment.

Variable	Univariate analysis (*n* = 268)		Multivariate analysis (*n* = 268)	
Independent	*P*	OR (95% CI)	*P*	OR (95% CI)
Epidural analgesia	0.046	4.872 (1.032–22.999)	0.072	4.478 (0.877–22.874)
**General information**				
Age (years)	0.136	0.887 (0.757–1.039)	0.191	0.890 (0.747–1.060)
Gestational age at delivery (weeks)	0.171	1.588 (0.819–3.079)		
BMI (kg/m^2^)	0.218	1.102 (0.944–1.287)	0.168	1.145 (0.944–1.389)
History of abnormal pregnancy	0.881	0.902 (0.233–3.495)		
Pregnancy with obstetric disease	0.928	0.908 (0.112–7.391)		
Cigarette, alcohol, and long-term medication use		NA		
**Source of health knowledge during pregnancy**				
Routine obstetric examination	0.117	0.161 (0.016–1.578)		
Maternity classes	0.108	0.367 (0.108–1.245)		
Internet resources or books	0.421	0.413 (0.048–3.550)		
Unplanned pregnancy	0.841	1.149 (0.296–4.466)		
Method of diabetes control (diet vs. medication)	0.047	4.125 (1.017–16.729)	0.029	5.277 (1.181–23.580)
Maternal blood glucose control during pregnancy	0.838	0.849 (0.177–4.077)		
Mode of delivery (Cesarean vs. vaginal)		NA		
Neonatal weight ≥3500 g	0.865	1.125 (0.290–4.371)		
**Duration of labor (min)**				
Stage 1	0.076	1.001 (1.000–1.003)		
Stage 2	0.583	0.995 (0.976–1.014)		
Stage 3	0.581	1.024 (0.942–1.113)		

Hosmer and Lemeshow goodness of fit (GOF) test; *χ*^2^ = 9.812, *df* = 8, *P* = 0.278. Cox and Snell pseudo-*R*^2^ = 0.045. Nagelkerke pseudo-*R*^2^ = 0.153.

We then repeated the univariate and multivariate analyses for factors influencing the rate of neonate transfer to the NICU for hypoglycemia treatment after PSM, using the same independent variables and parameters as described above (Table 5). From this multivariate analysis, only epidural analgesia was identified as a risk factor for neonatal hypoglycemia (OR: 12.526, 95% CI: 1.332–117.776; *P* < 0.05).

**Table 5:** Results of univariate and multivariate analyses of factors influencing the rate of neonate transfer to the NICU for hypoglycemia treatment after PSM.

Variable	Univariate analysis (*n* = 139)		Multivariate analysis (*n* = 139)	
Independent	*P*	OR (95% CI)	*P*	OR (95% CI)
Epidural analgesia	0.030	10.200 (1.255–82.875)	0.027	12.526 (1.332–117.776)
**General information**				
Age (years)	0.256	0.903 (0.757–1.077)	0.160	0.873 (0.722–1.055)
Gestational age at delivery (weeks)	0.128	1.729 (0.855–3.494)		
BMI (kg/m^2^)	0.188	1.106 (0.952–1.284)	0.104	1.202 (0.963–1.501)
History of abnormal pregnancy	0.520	0.592 (0.120–2.919)		
Pregnancy with obstetric disease	0.948	1.074 (0.125–9.219)		
Cigarette, alcohol, and long-term medication use		NA		
**Source of health knowledge during pregnancy**				
Routine obstetric examination	0.204	0.216 (0.020–2.292)		
Maternity classes	0.139	0.364 (0.095–1.388)		
Internet resources or books		NA		
Unplanned pregnancy	0.804	0.817 (0.164–4.055)		
Method of diabetes control (diet vs. medication)	0.556	0.613 (0.120–3.131)		
Maternal blood glucose control during pregnancy	0.017	6.429 (1.392–29.694)	0.053	5.384 (0.976–29.714)
Mode of delivery (Cesarean vs. vaginal)		NA		
Neonatal weight ≥3500 g	0.856	1.139 (0.279–4.652)		
**Duration of labor (min)**				
Stage 1	0.053	1.002 (1.000–1.003)		
Stage 2	0.638	0.996 (0.977–1.014)		
Stage 3	0.352	1.045 (0.953–1.146)		

Data were matched by using propensity score matching with 1 : 1 nearest neighbor matching. Hosmer and Lemeshow goodness of fit (GOF) test; *χ*^2^ = 9.992, *df* = 8, *P* = 0.266. McFadden's pseudo-*R*^2^ = 0.229. Cox and Snell pseudo-*R*^2^ = 0.109. Nagelkerke pseudo-*R*^2^ = 0.269.

**Figure 2:**
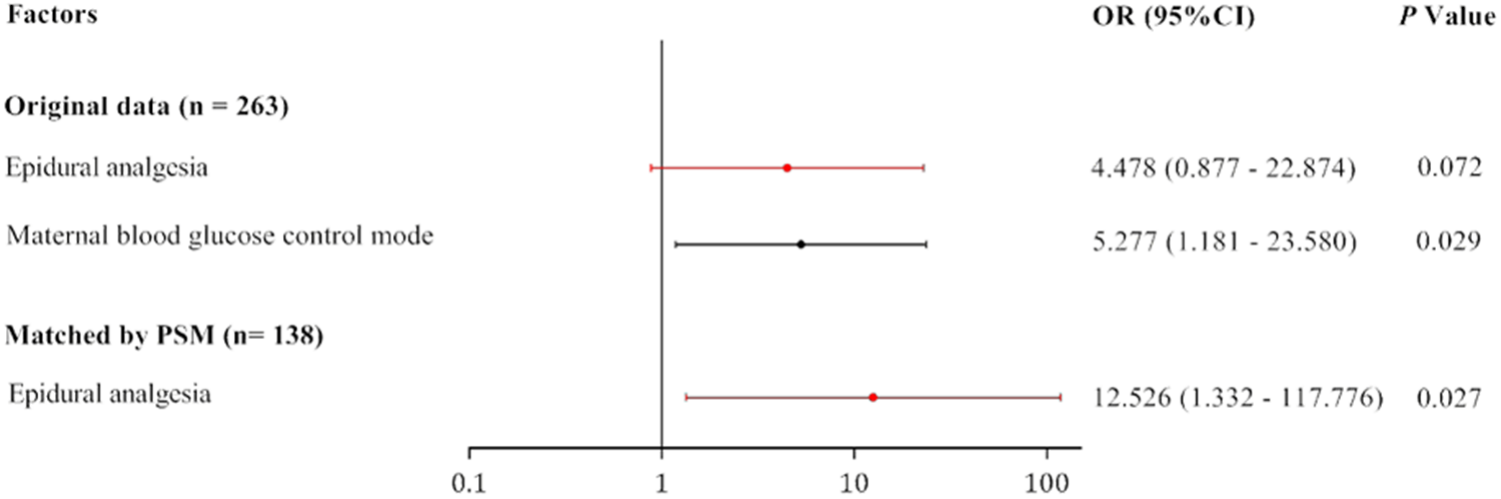
Independent influencing factor (IIF) on the rate of neonate transfer to the NICU for hypoglycemia treatment before and after PSM.

Thus, each analysis, with or without propensity score matching, identified one independent factor influencing the rate of neonate transfer to the NICU for hypoglycemia treatment was identified among parturients with GDM, but the factors differed with and without PSM (method of diabetes control without PSM and epidural labor analgesia with PSM; Figure 2).

### Model for predicting neonate transfer to the NICU for hypoglycemia treatment after delivery by parturients with GDM

**Figure 3:**
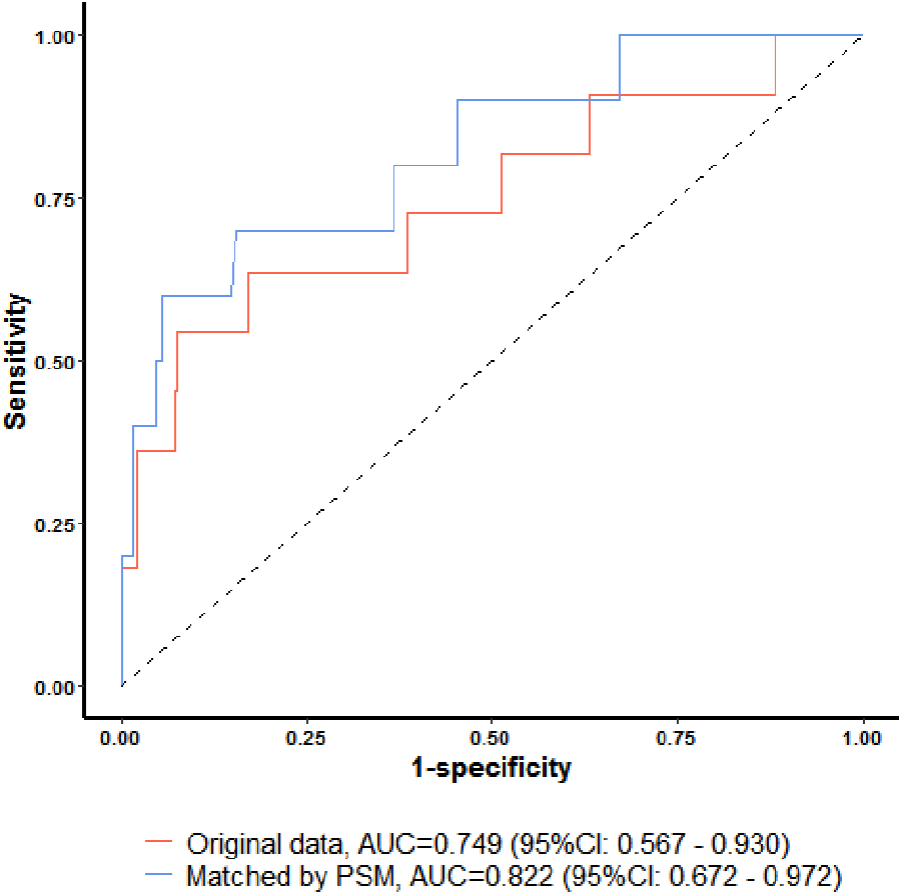
ROC curves on the rate of neonate transfer to the NICU for hypoglycemia treatment before and after PSM.

Two models based on the results of multiple logistic regression analysis were tested for their ability to predict the rate of neonate transfer to the NICU for hypoglycemia treatment. The model without PSM showed an AUC value of 0.749 (95% CI: 0.567–0.930), with a sensitivity of 0.545, specificity of 0.925, positive predictive value of 0.240, and negative predictive value of 0.979. The model with PSM showed an AUC value of 0.822 (95% CI: 0.672–0.972), with a sensitivity of 0.700, specificity of 0.852, positive predictive value of 0.240, and negative predictive value of 0.965 (Figure 3).

## Discussion

In the present study, the rate of neonatal admission to the NICU for hypoglycemia was higher in the epidural group (7.52%) than in the control group (1.48%; *P* < 0.05). Epidural analgesia and drug-based diabetes control were independent predictors of the rate of neonate transfer to the NICU for hypoglycemia. Labor pain was assessed at three time points and, as in previous studies, pain scores (NRS scores) in patients receiving epidural analgesia were significantly lower than those in the control group.

In this study, epidural labor analgesia significantly prolonged the first and second stages of labor (Table 2). As recommended by experts, the upper limit of epidural labor analgesia for primiparas was 4 h in the second stage of labor, and that for primiparas without epidural labor analgesia was 3 h. Thus, the durations of the two stages were still within the normal range (14). Previous studies have also reported that epidural labor analgesia can prolong the first and second stages of labor (15, 16), which is consistent with our findings. The incidence rates of pruritus, dizziness and urinary retention in the epidural group were higher than those in the control group, which may be related to the use of epidural opioids (17, 18). In the present study, parturients with failure of induction were not included. Thus, the rates of cesarean section (1% and 2%) were much lower than in these studies (16, 19), which is consistent with the findings of a previous study (20). Vaginal delivery rates in the epidural group remained similar to those in the control group. Therefore, it is encouraging that while epidural analgesia may prolong the first and second stages of labor and cause mild side effects, labor outcomes are not affected.

Diabetes control with medication and epidural analgesia were both identified as risk factors for neonate transfer to the NICU for hypoglycemia treatment among our GDM population. In women with GDM, diet is the first choice for blood glucose control, but if the effect is insufficient, drug control is the better choice. This means that women who require drug control are more likely to have experienced poor blood glucose control. Blachier et al. (21) found that neonatal hypoglycemia is associated with drug control of GDM and not associated with diet control of GDM, which is consistent with our results.

The pain and tension experienced during the perinatal period lead to a significant increase in the blood glucose concentration (22), and the fetal plasma catecholamine level can reach a very high level during delivery. This increase can be avoided by appropriate maternal pain relief and sympathetic block after epidural anesthesia (23). With the onset of pain relief, the catecholamine concentration decreases significantly, potentially resulting in an acute hypoglycemia attack in a diabetic parturient after administration of labor analgesia (10). Crites J et al. found that the serum concentrations of cortisol and adrenaline of mothers abruptly decreasedt after combined spinal–epidural anesthesia, which could trigger an acute hypoglycemic event (24). Indeed, epidural anesthesia reduces maternal stress hormone levels during labor (25). Studies have found that 6 h after delivery, the plasma cortisol level of parturients who received epidural anesthesia is lower than that in those who did not receive epidural anesthesia (26). The present study found that the incidence of hypoglycemia in newborns at 2 h after birth as well as the proportion of neonates with hypoglycemia requiring NICU treatment were higher in the epidural group than in the control group. These findings may be due to the fact that labor analgesia inhibits the stress response induced by pain and tension, thereby inhibiting the blood glucose concentration (27, 28). Beneventi et al. found that epidural analgesia reduces fetal cord arterial glucose and lactate levels in women with GDM (28). The pathophysiological basis of the high incidence of neonatal hypoglycemia in regional analgesia groups may be attributed to three factors: (1) increased utilization of glucose, (2) decreased gluconeogenesis, or (3) decreased glycogenolysis (29). As mentioned above, the labor pain has been proposed to be associated with a higher cortisol concentration in the neonate, which may thus prevent the occurrence of neonatal hypoglycemia.

However, M Westgren et al. found that epidural anesthesia reduced the maternal stress hormones at delivery but seemed to have little or no effect on the fetal endocrine stress hormones (25). In addition, Chen et al. reported that a higher maternal labor pain score and epidural anesthesia administration decreased the risk of neonatal hypoglycemia and proposed that maternal pain and epidural analgesia may have protective effects on neonatal hypoglycemia through other unknown mechanisms (30). The difference between the results of this study and ours may be related to the differences in participants' characteristics (age, parity, delivery mode), analytical methods (prospective, retrospective), etc. Further studies are needed to explore these discrepancies.

Our results showed that the incidence of neonatal hypoglycemia [<47 mg/dl (2.6 mmol/L)] was 11.54% in the epidural group and 5.93% in the control group at 1 h after delivery. A cohort study in an Israeli medical center reported an incidence of neonatal hypoglycemia of 12.1% among all newborns at 74 min after delivery (31). The lower rates in our study population may be due to differences in the subjects and deliveries. Overall, epidural analgesia is known to potentially lead to maternal hypoglycemia, which then leads to neonatal hypoglycemia. Because repeated or persistent severe hypoglycemia may cause damage to the central nervous system (32), it is necessary to monitor neonatal blood glucose levels closely after administration of epidural analgesia and apply timely treatment for neonatal hypoglycemia.

To our knowledge, this is the first prospective cohort study to investigate the effects of epidural analgesia on labor outcomes in pregnant women with GDM and their newborns. Although epidural analgesia was found to prolong the first and second stages of labor and increase the incidence of neonatal hypoglycemia and maternal side effects, it could reduce labor pain effectively. More research is needed to understand the association between epidural analgesia and hypoglycemia of neonates born to parturients with GDM.

This study has several limitations that should be considered. First, continuous monitoring of the blood glucose concentration of parturients was lacking, as invasive blood glucose monitoring was rejected by the patients and not strictly enforced. Second, group allocation was determined according to maternal choice. Third, our data were collected from a single center in Southern China. Most of the neonates were of the Han Chinese ethnic group, and therefore, the results of this study may not apply to neonates in other regions of China, or to other populations around the world.

## Conclusion

Epidural labor analgesia may be a protective factor against labor pain but a risk factor for neonatal hypoglycemia. Further studies with a larger sample size and longer follow-up time are needed to better characterize the effects of epidural labor analgesia on maternal outcomes and neonatal hypoglycemia.

## Data Availability

The original contributions presented in the study are included in the article/Supplementary Material, further inquiries can be directed to the corresponding author/s.
